# Stem cell-secreted 14,15- epoxyeicosatrienoic acid rescues cholesterol homeostasis and autophagic flux in Niemann–Pick-type C disease

**DOI:** 10.1038/s12276-018-0176-0

**Published:** 2018-11-14

**Authors:** Insung Kang, Byung-Chul Lee, Jin Young Lee, Jae-Jun Kim, Eun-Ah Sung, Seung Eun Lee, Nari Shin, Soon Won Choi, Yoojin Seo, Hyung-Sik Kim, Kyung-Sun Kang

**Affiliations:** 1 Adult Stem Cell Research Center, College of Veterinary Medicine, Seoul National University, Seoul, 08826 South Korea; 2 BK21 PLUS Program for Creative Veterinary Science Research and Research Institute for Veterinary Science, College of Veterinary Medicine, Seoul National University, Seoul, 08826 South Korea; 3 Biomedical Research Institute, Pusan National University School of Medicine, Pusan National University Hospital, Busan, Republic of Korea

## Abstract

We previously demonstrated that the direct transplantation of human umbilical cord blood-derived mesenchymal stem cells (hUCB-MSCs) into the dentate gyrus ameliorated the neurological symptoms of Niemann–Pick type C1 (NPC1)-mutant mice. However, the clinical presentation of NPC1-mutant mice was not fully understood with a molecular mechanism. Here, we found 14,15-epoxyeicosatrienoic acid (14,15-EET), a cytochrome P450 (CYP) metabolite, from hUCB-MSCs and the cerebella of NPC1-mutant mice and investigated the functional consequence of this metabolite. Our screening of the CYP2J family indicated a dysregulation in the CYP system in a cerebellar-specific manner. Moreover, in Purkinje cells, CYP2J6 showed an elevated expression level compared to that of astrocytes, granule cells, and microglia. In this regard, we found that one CYP metabolite, 14,15-EET, acts as a key mediator in ameliorating cholesterol accumulation. In confirming this hypothesis, 14,15-EET treatment reduced the accumulation of cholesterol in human NPC1 patient-derived fibroblasts in vitro by suppressing cholesterol synthesis and ameliorating the impaired autophagic flux. We show that the reduced activity within the CYP system in the cerebellum could cause the neurological symptoms of NPC1 patients, as 14,15-EET treatment significantly rescued cholesterol accumulation and impaired autophagy. We also provide evidence that the intranasal administration of hUCB-MSCs is a highly promising alternative to traumatic surgical transplantation for NPC1 patients.

## Inherited metabolic disease: Restoring motor function by reducing cholesterol

An acid secreted by stem cells can reduce the excess cholesterol caused by a genetic metabolic disorder. Niemann–Pick type C disease is a rare, inherited condition that causes defective muscular development and progressive neurological degeneration. A key disease mechanism is the excessive accumulation of cholesterol within cells. Kyung-Sun Kang at Seoul National University, South Korea, and co-workers have demonstrated that a metabolite molecule called 14,15-epoxyeicosatrienoic acid (14,15-EET) derived from stem cells from human umbilical cord blood significantly reduced cholesterol in Neimann-Pick Type C mouse models and human cell samples. The team administered the stem cell therapy non-invasively via the nose, and observed significant improvements in motor function in the mice. Experiments in both animals and cells showed that the treatment resulted in reduced cholesterol levels and the correction of defective signalling within cells.

## Introduction

Niemann–Pick type C (NPC) disease is an inherited lipid storage disorder, with an estimated incidence of 1:20,000 to 1:150,000 live births. The majority of NPC patients have mutations in the *NPC1* gene (95% of cases), while 5% of cases are associated with a defect in the *NPC2* gene^1^. The dysfunction of NPC proteins leads to a defect in intercellular cholesterol trafficking, characterized by the impaired exit of cholesterol from late endosomes/lysosomes (LE/L)^2^. Progressive neurodegeneration with a specific loss of cerebellar (CB) Purkinje cells is one of the primary indicators of NPC, which results in the development of several neuromuscular symptoms, such as ataxia, dysarthria, and dysphagia, during growth^3^.

The excessive accumulation of cholesterol in endolysosomes is considered to be a major pathogenic mechanism of NPC disease^4^. Several strategies to reduce cholesterol levels in NPC disease treatment have been attempted. Previously, NPC1-mutant mice treated with hydroxypropyl-β-cyclodextrin in primary cultures of neurons and glial cells had significantly improved levels of unesterified cholesterol in LE/L^5^. In addition, we previously demonstrated that treatment with valproic acid, a histone deacetylase inhibitor, reduced cholesterol levels in neural stem cells from NPC1-mutant mice^6^. However, these approaches lack mechanistic studies; therefore, their therapeutic effects have not been determined.

To date, the significant potential of using mesenchymal stem cells (MSCs) for the treatment of neurological disorders has been addressed. The direct transplantation of bone marrow-derived MSCs (BM-MSCs) into the cerebella of NPC1-mutant mice reduced both astrocytic and microglial activation and increased Purkinje cell survival, thereby improving the clinical outcome in mice^7–9^. Similarly, we reported that the hippocampal transplantation of human umbilical cord blood-derived MSCs (hUCB-MSCs) not only activated endogenous neurogenesis in the dentate gyrus but also protected Purkinje cells and the motor function of NPC1-mutant mice by reducing the intracellular cholesterol deposits^10^. MSCs may be specifically manipulated to transdifferentiate into other cell types, which enables them to replace lost host cells; however, they also have multifunctional roles in immunomodulation, intrinsic stem/progenitor cell stimulation, tissue regeneration, and angiogenesis, largely based on their paracrine activities. Therefore, elucidating the specific trophic factors that underlie the therapeutic effects of MSCs could uncover benefits of MSC application in other pathological conditions, as well as enhance the therapeutic capacity of MSCs.

Due to the presence of the blood–brain barrier, direct cell transplantation into the target region is the most frequently used method within the central nervous system; however, a less invasive route is preferable for further clinical applications. Recent studies have evaluated the nasal system as an alternative cell delivery route to the brain. Intranasally applied MSCs have been shown to migrate through the cribriform plate and settle in the brain tissue via the olfactory and trigeminal pathways^11^. Importantly, MSCs migrate to various regions, such as the cortex, hippocampus (HP), striatum, cerebellum, brain stem, and spinal cord^12^, which implies that stem cell delivery via nasal passages may enable the entire central nervous system to be targeted.

As an extension of our previous study, we assessed the therapeutic capacity of hUCB-MSCs on NPC1 disease using human NPC1 fibroblast (FB NPC1) (in vitro) and NPC1-mutant mouse (in vivo*)* models. The nasal delivery of hUCB-MSCs could reduce the loss of Purkinje cells in the NPC1-affected cerebellum and delay motor dysfunction. In this study, we focused on the potential role of hUCB-MSCs to address the impaired cholesterol trafficking associated with NPC1 disease, as hUCB-MSCs appear to decrease cholesterol accumulation both in vivo and in vitro. Interestingly, we found that 14,15-epoxyeicosatrienoic acid (14,15-EET), an arachidonic acid metabolite synthesized by cytochrome P450 (CYP) epoxygenases of the 2C and 2J subfamilies^13^, mediated the cholesterol-regulating role of hUCB-MSCs by triggering the autophagic pathway.

## Materials and methods

### Isolation and maintenance of hUCB-MSCs

hUCB-MSC isolation and culture were performed as previously described^14,15^. The Seoul City Borame Hospital Cord Blood Bank provided the hUCB samples. Samples from term and preterm deliveries were harvested at the time of birth with informed consent from the mothers. This work was approved by the Borame Hospital Institutional Review Board and Seoul National University (IRB No. 1608/001-021). Blood samples were processed within 24 h of collection. After mixing the UCB samples with HetaSep solution (StemCell Technologies, Vancouver, Canada) at a ratio of 5:1, mononuclear cells were separated from the UCB using a Ficoll-Paque TM PLUS (Amersham Bioscience, Uppsala, Sweden). Cells were seeded on plates at a density of 2 × 10^6^ cells/cm^2^ in KSB-3 Complete Medium (Kangstem Biotech, Seoul, Korea) supplemented with 10% fetal bovine serum (Gibco BRL, Grand Island, NY, USA), 100 I/ml penicillin, and 100 mg/ml streptomycin.

### Animal model

Pairs of heterozygous Balb/c NPC^*+/−*^ mice were purchased from Jackson Laboratories (Bar Harbor, MA, USA). Through breeding, the wild-type control (WT; NPC^*+/+*^) and NPC1-knockout (NPC1; NPC^*−*/*−*^) mice were generated. Genotyping was carried out as previously described^16^. An authorized animal facility maintained all animals under strict management. Every experiment was performed in accordance with the regulations of the Laboratory Animals Resources (SNU IACUC Approval No: SNU-130717-3-1, Title of proposal: therapeutic approaches for NPC mice; Institutional Animal Care and Use Committee, Seoul National University, Korea).

### hUCB-MSC administration via the nasal route in vivo

At 4 weeks of age, anesthetized animals were placed in a supine position, and 3 µl of hyaluronidase (total 100 U; Sigma-Aldrich Chemical Co.) in phosphate-buffered saline (PBS) was applied to each nostril prior to hUCB-MSC or vehicle administration^11^. Sterile PBS or 1 × 10^6^ hUCB-MSCs were subsequently applied to each nostril (6 µl per nostril, 3 µl each time). This treatment was repeated three times weekly.

### Rotarod test

To coordinate the motor function ability, we used a rotarod treadmill (7650 Accelerating model, Ugo Basile Biological Research Apparatus, Comerio, Italy). At 4 weeks of age, the mice were trained for 2 weeks prior to having their motor function tested once per week from 6 to 8 weeks of age at a speed of 10 rpm and a maximum duration of 180 s. The representative record of each subject was adopted as the mean performance time from four attempts^17^.

### Fluorescent immunostaining

The brains were perfused with 0.1 M PBS (pH 7.4) followed by 4% paraformaldehyde in 0.1 M PBS. They were then isolated and soaked in 4% paraformaldehyde in 0.1 M PBS overnight for post-fixation. For the immunohistochemistry analysis, the brain tissues were transferred to a mold filled with an infiltration mixture (OCT compound; Sakura Finetek, Tokyo, Japan) and were maintained at −70 °C overnight until they were cryosectioned at 20 µm thick on a cryostat (CM 3050, Leica, Wetzlar, Germany) and used for Nissl staining. To compare the levels of accumulated cholesterol, the sections were stained with filipin as previously described^16^. The sections were subsequently washed with PBS and incubated with calbindin (CBD) primary antibody (Abcam, Cambridge, MA, USA), followed by being extensively washed with PBS and incubated with anti-rabbit secondary antibody conjugated with Alexa Fluor 488 (Molecular Probes, Eugene, OR, USA) for 1 h. Hoechst 33238 (1 µg/ml, Sigma-Aldrich) staining was used to visualize the cell nuclei. Images were captured and merged with a confocal microscope system (Eclipse TE200, Nikon, Nagano City, Japan).

For immunocytochemistry (ICC), human FB was plated on 12-mm coverslips with 1 × 10^4^ cells/slip. After chemical treatment or transwell co-culture, cells were fixed with 4% paraformaldehyde for 5 min and permeabilized with 0.05% Triton X-100. Blocking was performed in 5% normal goat serum. After staining with primary antibodies, the samples were stained with Alexa 488 antibodies or Alexa 594-conjugated secondary antibodies (Molecular Probes, Eugene, OR, USA), and 4′,6-diamidino-2-phenylindole (DAPI) (Zymed Laboratories Inc.) was used for nuclear counterstaining.

### Immunoblotting

The brains of 8-week-old mice were removed, and the cerebella were separated from the whole brains. Each cerebellum was homogenized with lysis buffer (Pro-PREP, Inton Biotechnology, Korea), and the protein concentration was quantified using the DC Assay Kit (Bio-Rad, Berkeley, CA, USA). For Western blot analysis, equal amounts of protein (20 µg) were separated via 10–15% sodium dodecyl sulfate-polyacrylamide gel electrophoresis (SDS-PAGE) and transferred to nitrocellulose membranes. The membranes were blocked with 3% bovine serum in a Tris-buffered saline with Tween (TBST: 20 mM Tris-HCl (pH 7.6), 137 mM NaCl, 1% Tween-20) and incubated with primary antibody (LC3, Novus). After incubating with the secondary antibody according to the manufacturer’s specifications (horseradish peroxidase-conjugated antibody (1:2000; Invitrogen, Carlsbad, CA, USA; G21234)), proteins were detected with an Enhanced Chemiluminescence Detection Kit (Amersham Pharmacia Bioteck, Amersham, UK). The relative band intensities were determined using the NIH ImageJ software version 1.51f.

### Gene expression analysis using quantitative real-time PCR

Total RNA was extracted from cells using TRIzol reagent (Invitrogen, Carlsbad, CA, USA) according to the manufacturer’s instructions. Next, 1 µg of RNA was reverse transcribed to complementary DNA using the Superscript First-Stand Synthesis System (Invitrogen, Carlsbad, CA, USA). The relative messenger RNA (mRNA) levels were determined using SYBR Green PCR Master Mix (Applied Biosystems, Foster City, CA, USA) with an ABI 7300 sequence detection system and the supplied software. The level of expression for each gene was normalized to that of the housekeeping control gene *GAPDH*. The gene expression levels were measured at least three times.

### ELISA analysis

The amount of secreted 14,15-EET in the supernatants from the hUCB-MSC culture was measured using a 14,15-EET/DHET ELISA (enzyme-linked immunosorbent assay) Kit (cat. no DH2R; Detroit R&D) according to the manufacturer’s instructions.

### GPF-expressing hUCB-MSCs

To make GPF-expressing hUCB-MSCs, 5 × 10^5^ hUCB-MSCs were counted and seeded into 6-well plates. When the cell cultures were 70% confluent, polybrene-facilitated transfection was performed using a viral vector that encoded enhanced green fluorescent protein (70 µl of vector, 0.3 µl of polybrene/cell). The culture medium was replaced after 24 h. For selection, after 2 additional days of cell culture, the media were replaced with media that contained neomycin (600 µg/ml).

### Flow cytometric analysis for cell tracking

Twenty-four hours after intranasal delivery of GPF-expressing hUCB-MSCs, the mice were sacrificed, and the brain was collected. The mice without hUCB-MSCs treatment served as the controls. Brain regions were separated and mechanically dissociated by repeated manual pipetting. Following sufficient PBS washing and the removal of red blood cells with red blood cell lysis buffer (Roche, Basel, Switzerland), the tissue was single celled with 0.05% trypsin solution. Flow cytometry analysis was performed on a FACSCaliber using Cell Quest software (Becton Dickinson, Franklin Lakes, NJ, USA).

### Quantification and statistical analysis

During histological assessment, the sections were analyzed and anatomically matched between animals. The relative immune densities of each signal after Western blotting and immunostaining were measured using NIH ImageJ software version 1.63. The results are shown as the mean ± SD for independent experiments. Statistical analysis of the significance was calculated using Graphic Pad Prism (v5.0, GraphPad) with unpaired Student’s *t* test and one-way analysis of variance followed by Bonferroni post hoc analysis.

## Results

### Tracking the migration of GFP-expressing hUCB-MSCs delivered intranasally in vivo

**Fig. 1:**
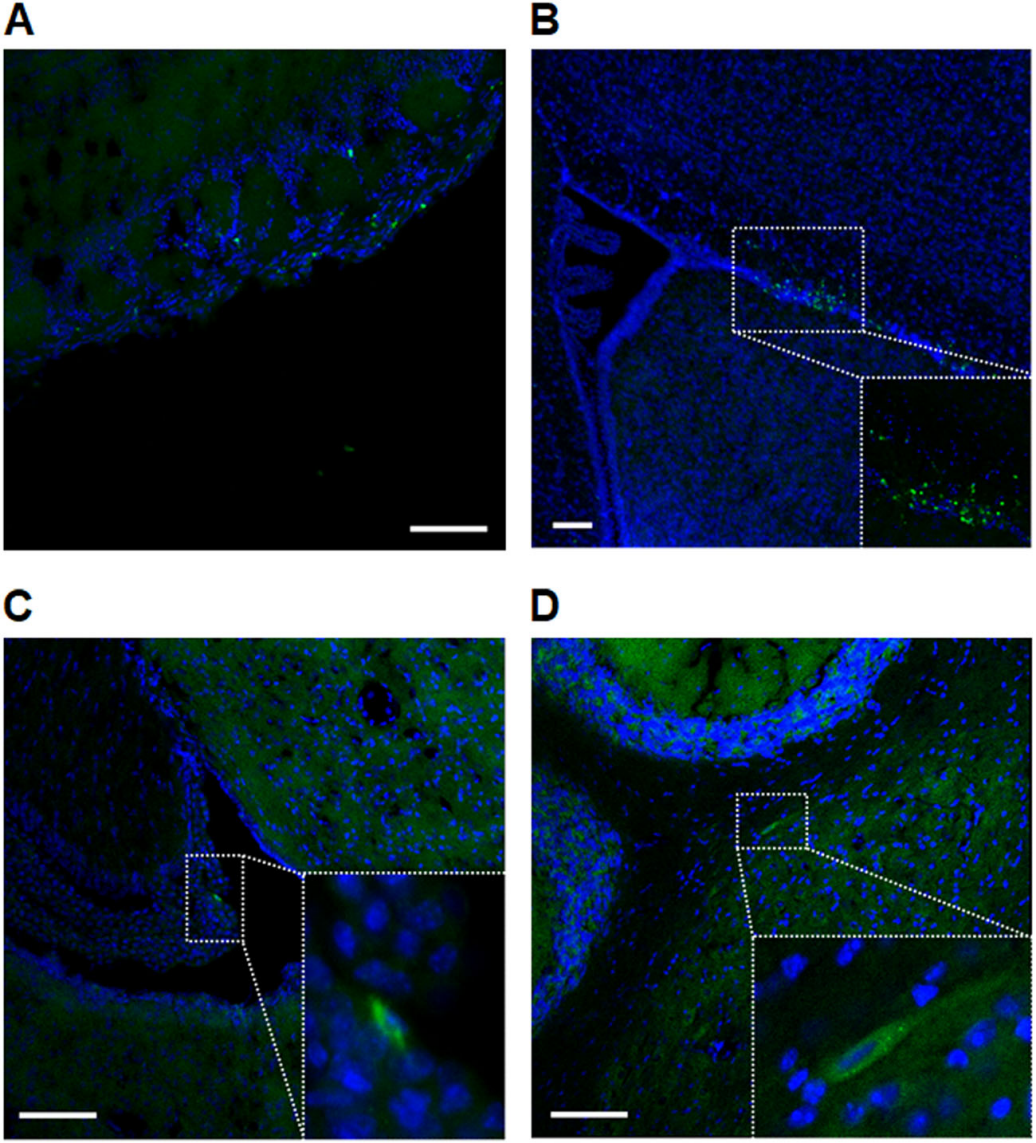
hUCB-MSC location in the brain after intranasal administration. **a** Increased density of GFP-expressing hUCB-MSCs within the glomerular layer of the olfactory bulb. **b** A different distribution pattern, focused throughout the rostral migratory stream, was examined. **c** No dense GFP-expressing hUCB-MSC was exhibited in the hippocampus area; however, single cells that expressed GFP were visible and magnified. **d** The deep cerebellar nuclei also show single hUCB-MAC-expressing GFP and were also magnified. Scale bars = 50 μm

To evaluate the migration ability, we first produced GFP-expressing hUCB-MSCs and then delivered the cells to mice via the intranasal route. After administration, the GFP signals were mainly identified in the glomerular layer of the olfactory bulb (OB) (Fig. 1a). In addition to the OB, cells were distributed throughout the rostral migratory stream (RMS) (Fig. 1b). Moreover, we could detect single GFP-positive cells in the HP region (Fig. 1c) and the CB nuclei (Fig. 1d). Quantification of the GFP signal indicated that hUCB-MSCs were mainly distributed in the OB region compared with other brain regions (subventricular zone (SVZ), HP, and CB) (Fig. S1A). Through flow cytometric analysis, we confirmed the distribution of GFP-expressing hUCB-MSCs. The cells were mostly distributed in the OB, and the total percentage of GFP-expressing hUCB-MSCs detected per OB was 1.23% (Fig. S1B).

### Behavioral performance was improved in NPC1-mutant mice after intranasal delivery of hUCB-MSCs

**Fig. 2:**
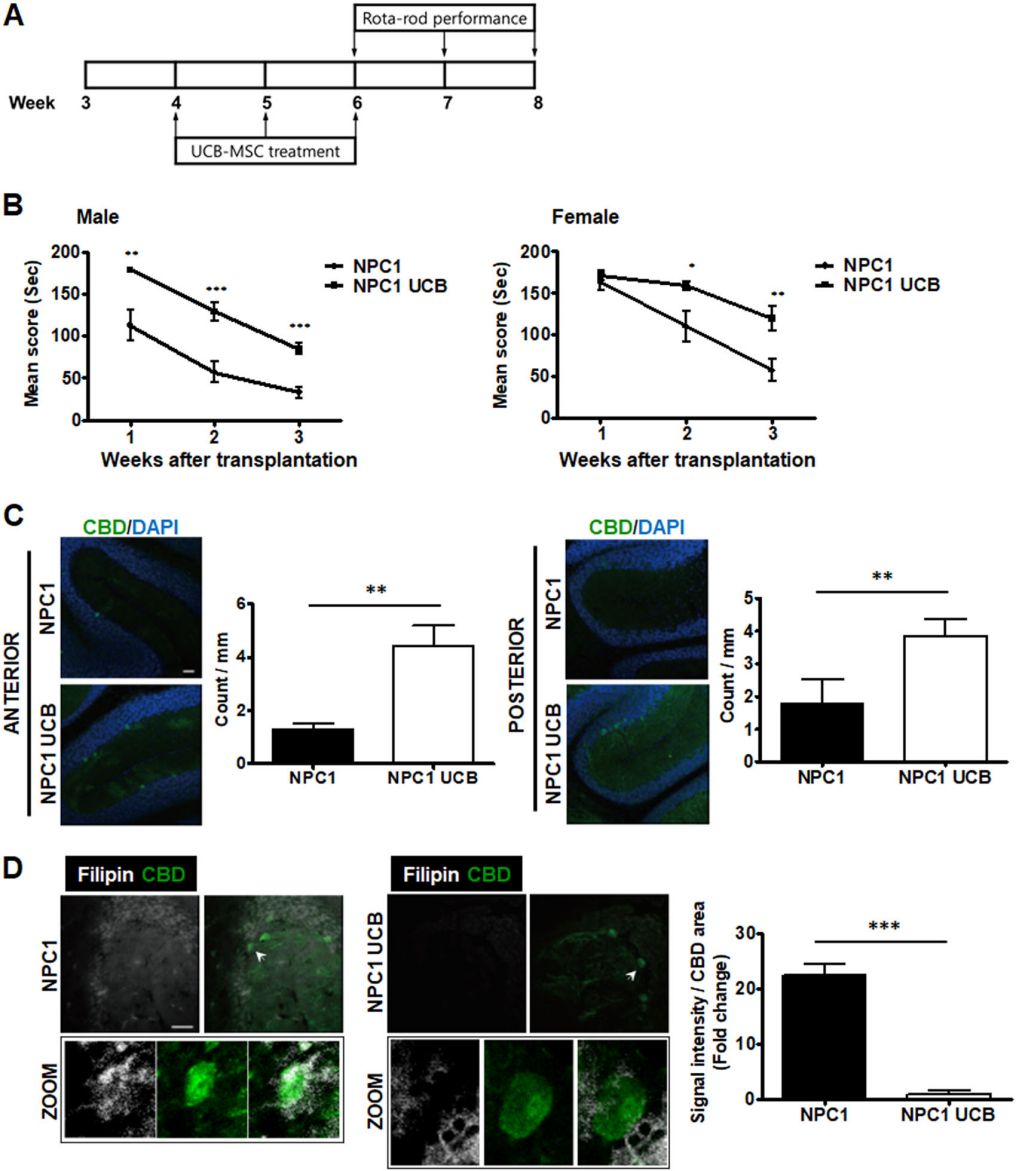
Motor function and Purkinje cell survival rate in NPC1-mutant mice after intranasal administration of hUCB-MSCs. **a** Transplantation scheme for intranasal delivery of hUCB-MSCs in NPC1-mutant mice. **b** Compared with NPC1-mutant mice (NPC1), intranasal delivery of UCB-MSCs (NPC1-UCB) improves performance on the rotarod test in both males (NPC1 *n* = 9, NPC1-UCB *n* = 9) and females (NPC1 *n* = 6, NPC1-UCB *n* = 7). **c** Representative immunohistochemical images showing the distribution pattern of calbindin-positive Purkinje cells within the anterior and posterior regions of the cerebella of NPC1-mutant mice (NPC1) and hUCB-MSC administered NPC1-mutant mice (NPC1-UCB). The number of calbindin-expressing cells is indicated. Posterior region of the cerebella was also examined. **d** Cholesterol accumulation pattern in the Purkinje cells assessed by immunohistochemistry. NPC1-mutant mouse cerebella labeled with anti-calbindin show co-localization with the cholesterol detection marker filipin. hUCB-MSC treatment in NPC1-mutant mice results in less accumulation of filipin in the Purkinje cells. Arrowhead indicates magnified Purkinje cells. All data represent the mean ± SD. Scale bars = 50 μm (**a**, **b** share the same scale bar; **c**, **d** share the same scale bar). *P < 0.05; **P < 0.01; ***P < 0.001

NPC1 mutation in mice leads to an age-dependent impairment of motor tasks due to the loss of Purkinje cells^17^. To evaluate the therapeutic effects of intranasal delivery of hUCB-MSCs in NPC1-mutant mice, we performed a rotarod test, a widely used behavioral test to assess motility performance (Fig. 2a). The NPC1-mutant mice that received hUCB-MSCs intranasally (NPC1-UCB) showed an improved performance in the rotarod test. On average, the NPC1-UCB mice could endure the machine for 175.2 ± 2.85, 142.0 ± 7.61, and 99.92 ± 8.25 s at 1, 2, and 3 weeks post injection, respectively, whereas the NPC1-mutant mice treated with the vehicle (NPC1) could last for a shorter amount of time (137.7 ± 12.54, 85.92 ± 13.02, and 44.63 ± 6.92s, respectively) (Fig. S2). When evaluating mates, both male and female NPC1-UCB mice showed improved performances (Fig. 2b).

### Intranasal delivery of hUCB-MSCs improved CB Purkinje cell survival and cholesterol accumulation in NPC1-mutant mice

Clinically manifested, CB involved tremor and ataxia comprise one of the hallmarks of NPC1 disease in both humans and animal models^18^. Degeneration and the loss of Purkinje cells are well-established causes of the neurological symptoms related to the dysfunctional motor ability^19^. We assumed that improved motor function in NPC1-UCB mice would be a result of increased Purkinje cell survival in the cerebellum. Therefore, we counted the number of CBD-positive cells in the Purkinje cell layer of 8-week-old NPC1-mutant mice. As shown by immunohistochemistry, the NPC1-UCB mice exhibited a remarkable preservation of Purkinje cells compared to the non-treated mice. Compared to the NPC1-mutant mice, the total number of CBD-positive cells in the NPC1-UCB mice increased 3.46-fold and 2.17-fold in the anterior and posterior regions, respectively (Fig. 2c). Nissl staining also showed that the number of intact Purkinje cells increased 5.75-fold in the anterior region (Fig. S3A) and 2.1-fold in the posterior region following hUCB-MSC treatment (Fig. S3B).

To determine the cholesterol levels within the Purkinje cells, filipin and CBD double staining of cerebellum samples from NPC1-mutant mice and NPC1-UCB mice was performed. The NPC1-mutant mice displayed a co-localized staining pattern of filipin and CBD (Fig. 2d). In contrast, the NPC1-UCB mice displayed decreased filipin staining, specifically in the CBD-positive area (22-fold less), which indicates that intranasally delivered hUCB-MSCs could reduce the accumulation of unesterified cholesterol.

### CYP2J subfamilies are downregulated in NPC1-mutant mouse cerebellum

A previous study demonstrated that the overexpression of CYP2J2 significantly attenuated high-fat-diet-induced changes in triglyceride levels and cholesterol levels in the plasma and livers of mice. CYP2J2 overexpression leading to increased synthesis of 14,15-EET attenuated the lipid accumulation in free fatty acid-induced HepG2 cells, LO2 cells, and HUVECs^20^. Because NPC1 patients exhibit increased levels of fatty acids in the late endosomal/lysosomal system^21^, we hypothesized that 14,15-EET could be a therapeutic candidate for NPC1 patient treatment.

**Fig. 3:**
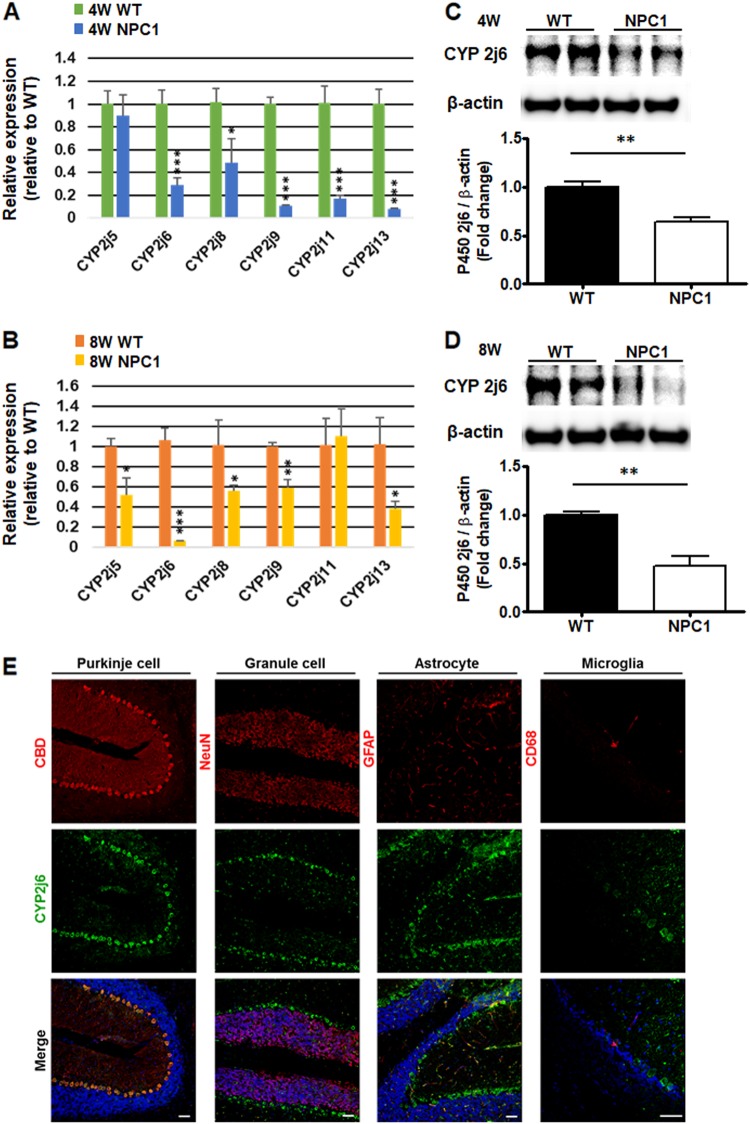
Expression of CYP2J subfamilies in WT and NPC1-mutant mice. **a** The expression levels of CYP2J subfamily genes were quantified at 4 weeks of age and **b** 8 weeks of age from WT and NPC1-mutant mouse cerebella (NPC1). **c** Immunoblot analysis with anti-CYP2J6 antibodies at 4 weeks of age and **d** 8 weeks of age from WT and NPC1-mutant mouse cerebella. **e** Immunohistochemistry analysis of the co-localization of CYP2J6 (CYP2J6; green) with Purkinje cells (calbindin; red), granule cells (NeuN; red), astrocytes (GFAP; red), and microglia (CD68; red) in WT cerebella. Nuclei were counterstained with DAPI. Experiments were conducted in triplicate as the mean ± SD. Scale bars = 50 μm. *P < 0.05; **P < 0.01; ***P < 0.001

Given that CYP2J can modulate lipid accumulation, we first screened the mRNA expression of the CYP2J subfamily of the cerebellum. The real-time PCR results showed that the mRNA levels of *Cyp2J5*, *Cyp2J6*, *Cyp2J8*, *Cyp2J9*, *Cyp2J11*, and *Cyp2J13* were expressed in the mouse cerebellum. The mRNA levels of *Cyp2J6*, *Cyp2J8*, *Cyp2J9*, *Cyp2J11*, and *Cyp2J13* were significantly reduced in the NPC1-mutant mice at 4 weeks of age (Fig. 3a), and *Cyp2J5*, *CypJ6*, *Cyp2J8*, *Cyp2J9*, and *Cyp2J13* were reduced at 8 weeks of age (Fig. 3b). At both 4 and 8 weeks of age, the mRNA level of *Cyp2J6* showed the most dramatic reduction. In addition, Western blot analysis of the cerebellum lysate confirmed that the expression density of Cyp2J6 in the NPC1-mutant mice was significantly reduced compared with the WT controls at 4 and 8 weeks of age (Fig. 3c, d).

To further explain the reduced CYP2J6 level in the NPC1-mutant mouse cerebellum, we assessed the expression of CYP2J6 in a region-specific manner. The cerebellum of an 8-week-old WT mouse was stained with CYP2J6, and the expression was strongest in the outer layer of the cerebellum lobes. CBD, a Purkinje cell marker, was co-stained with CYP2J6, and co-expression was demonstrated. Antibody staining for NeuN, which marks differentiating granule cells, or GFAP, which marks astrocytes, and CD68, which is a marker for microglia, were also co-stained with CYP2J6. The granule cells, astrocytes, or microglia did not express high levels of CYP2J6 (Fig. 3e). Altogether, CYP2J6 was mainly expressed from Purkinje cells rather than granule cells, astrocytes, or microglia.

### 14,15-EET is dysregulated in the NPC1-mutant mouse CB region

**Fig. 4:**
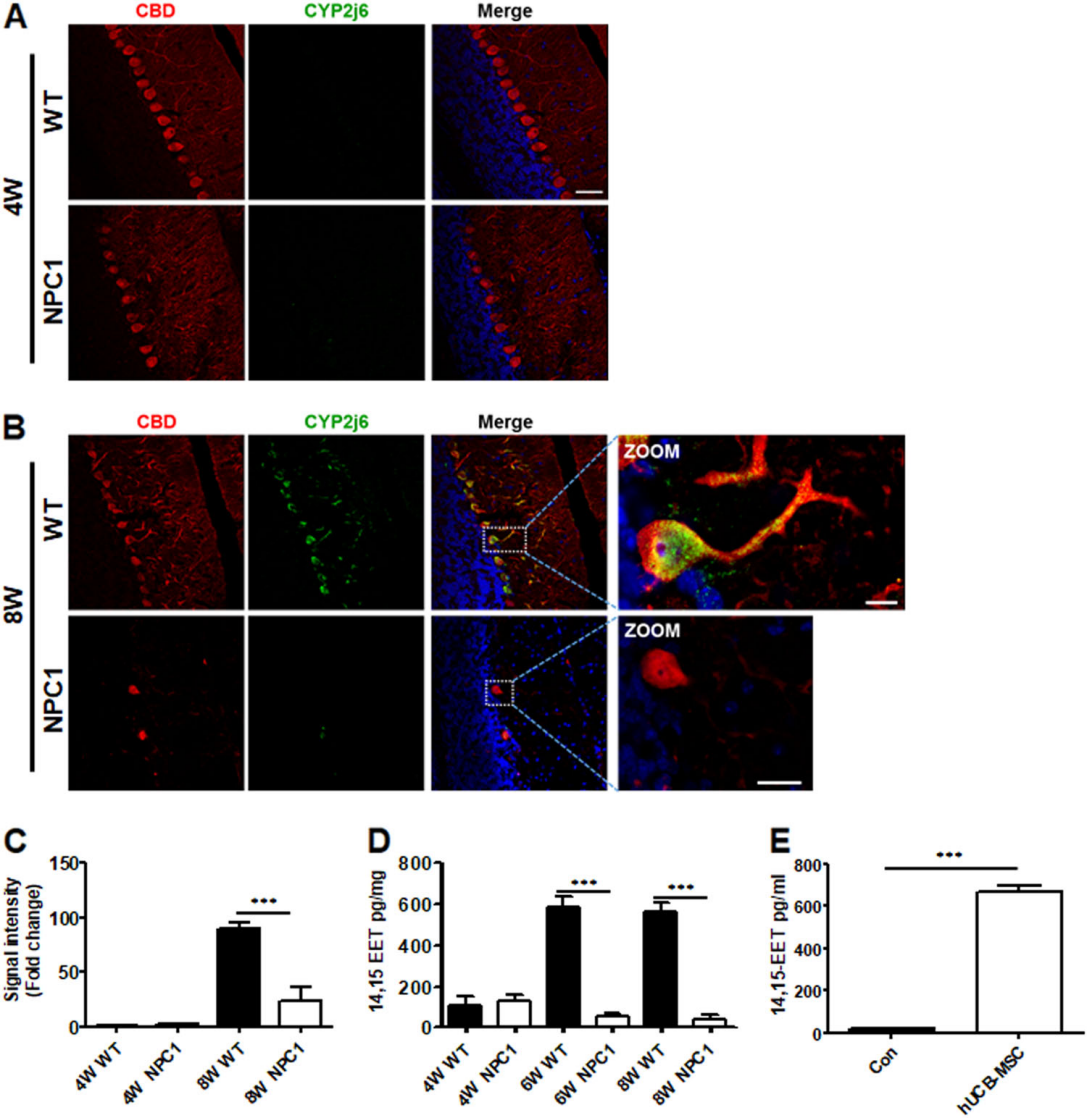
Specific expression of CYP2J6 in Purkinje cells in NPC1-mutant mice and 14,15-EET expression in WT, NPC1-mutant mouse cerebella, and hUCB-MSCs. **a** Comparison of immunocytochemistry analysis of the co-localization of CYP2J6 (CYP2J6; green) with Purkinje cells (calbindin; red) at 4 weeks of age and **b** 8 weeks of age in WT and NPC1-mutant mouse cerebella (NPC1). **c** Relative expression of CYP2J6 shows decreased expression of CYP2J6 at 8 weeks of age in NPC1-mutant mouse cerebella. **d** Through ELISA, 14,15-EET was analyzed at 4 weeks, 6 weeks, and 8 weeks of age from WT and NPC1-mutant mouse cerebella. **e** 14,15-EET in the free media and the supernatants of hUCB-MSC culture media were collected and compared by ELISA. All data represent the mean ± SD. Scale bars = 50 μm (**a**, **b** share the same scale bar; **c**, **d** share the same scale bar). ***P < 0.001

We compared the CB region of the WT and NPC1-mutant mice at 4 and 8 weeks of age. At 4 weeks of age, there was no detection of CYP2J6 in both the WT and NPC1-mutant mouse Purkinje cell layer (Fig. 4a). However, the expression of CYP2J6 showed a substantial increase in the Purkinje cells of the WT mice at 8 weeks of age. Compared with the WT, the NPC1-mutant mice presented a significantly reduced number of Purkinje cells, and the CYP2J6 expression was notably decreased in the Purkinje cells. Zoom images confirmed reduced CYP2J6 expression in the cytoplasm and neuron branch of the Purkinje cells (Fig. 4b, c).

To investigate the functional effects of NPC1 disease-associated neuron loss on CYP-mediated eicosanoid metabolism, we quantified the CB eicosanoid levels in the NPC1-mutant mice. CYP2J subfamilies catalyze the formation of 14,15-EET. When 14,15-EET was measured from the cerebellum at 8 weeks and compared between the WT and NPC1-mutant mice, 14,15-EET was significantly decreased in the NPC1-mutant mice. At 6 weeks, the results were similar, as the 14,15-EET levels were reduced in the NPC1-mutant mouse cerebellum. However, at 4 weeks, the 14,15-EET levels were low in the WT and NPC1-mutant mouse cerebellum samples (Fig. 4d). In our previous study, we determined whether specific cytokines were released from hUCB-MSCs^16^. To confirm the secretion of 14,15-EET from hUCB-MSCs, we analyzed the concentration of 14,15-EET from the cell-culture media of hUCB-MSCs using an ELISA (Fig. 4e). Interestingly, we identified a significant level of 14,15-EET, which indicates that hUCB-MSCs are a secretion source for 14,15-EET.

### The effect of hUCB-MSC in NPC1 disease is regulated by 14,15-EET

**Fig. 5:**
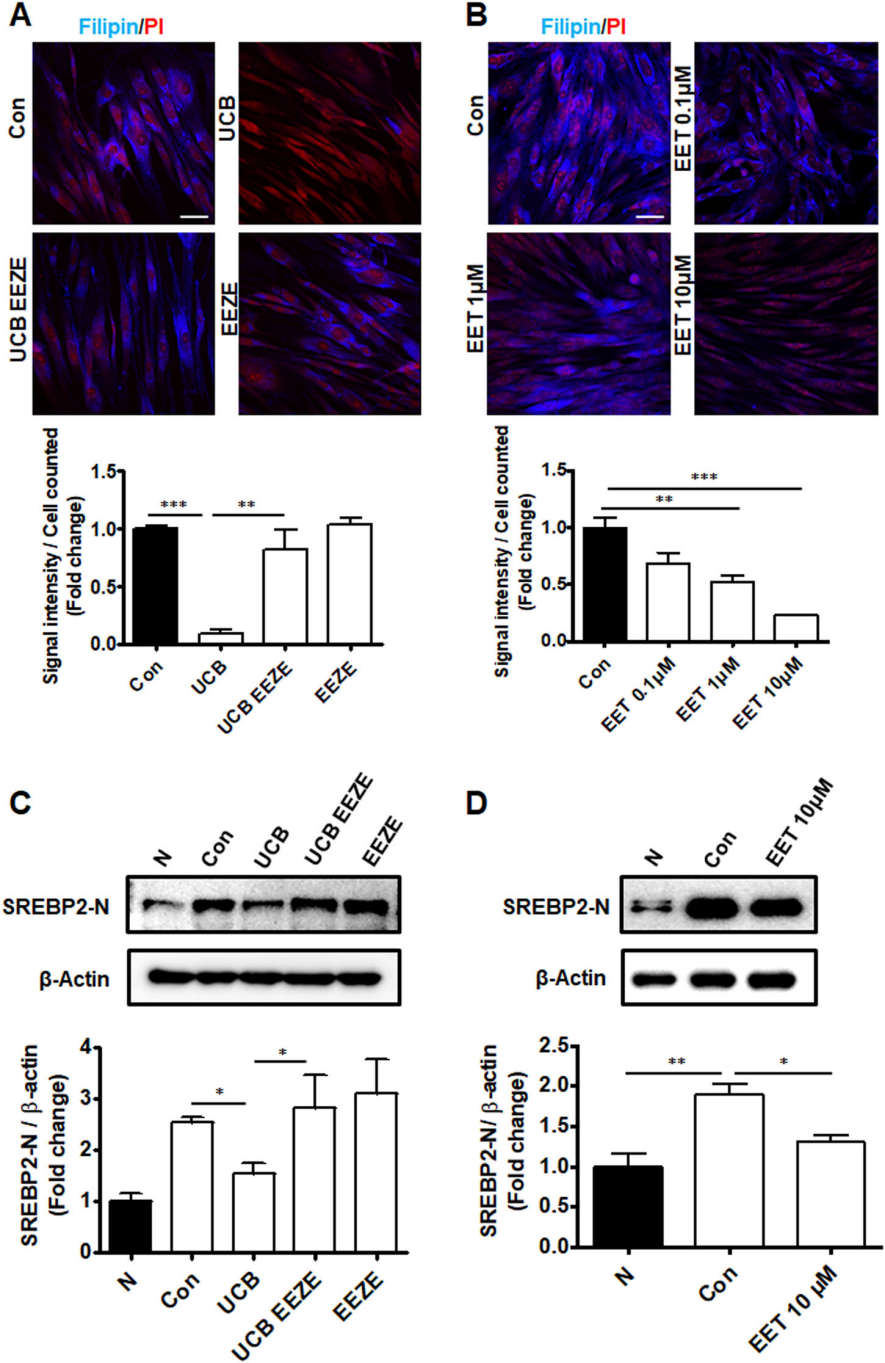
14,15-EET rescues cholesterol accumulation. **a** Representative in vitro fluorescence image of NPC1 patient fibroblasts (Con) labeled with PI and the cholesterol detection marker filipin. Co-culture with hUCB-MSCs (UCB) significantly reduces the accumulation of cholesterol in NPC1 patient fibroblasts. When EEZE is treated during the co-culture of hUCB-MSCs and NPC1 patient fibroblasts (UCB EEZE), cholesterol accumulation can be observed in the fibroblast. A single treatment of EEZE in NPC1 patient fibroblasts has no effect on cholesterol accumulation (EEZE). The intensity of filipin was quantified and normalized to non-treated NPC1 patient fibroblasts. **b** 14,15-EET was administered to NPC1 patient fibroblasts (Con) in three independent doses (0.1, 1, and 10 μM) and stained with filipin. The intensity of filipin was quantified and normalized to non-treated NPC1 patient fibroblasts. **c** Immunoblot analyses with anti-SREBP2 and anti-actin antibodies in normal fibroblasts (N), NPC1 patient fibroblasts (Con), hUCB-MSC-treated NPC1 patient fibroblasts (UCB), hUCB-MSC-co-treated and EEZE-co-treated NPC1 patient fibroblasts (UCB EEZE), and EEZE-treated NPC1 patient fibroblasts (EEZE). **d** Immunoblot analyses with anti-SREBP2 normalized by anti-actin antibodies in normal fibroblasts (N), NPC1 patient fibroblasts (Con), and 14,15-EET-treated NPC1 patient fibroblasts (10 μM), (EET 10 μM). The cleaved nuclear forms of SREBP2 are shown (SREBP2-N). Scale bars = 50 μm. All data represent the mean ± SD. *P < 0.05; **P < 0.01; ***P < 0.001

To determine the effects of hUCB-MSC-derived 14,15-EET on the cellular cholesterol levels in FB NPC1 cells, 14,15-epoxyeicosa-(*Z*)-enoic acid (EEZE), a 14,15-EET antagonist, was applied to an indirect co-culture of UCB-MSCs and FB NPC1 cells. As expected, the cholesterol accumulation was decreased when FB NPC1 cells were co-cultured with hUCB-MSCs. Compared to the FB NPC1 cells, the accumulated cholesterol in the FB NPC1 cells did not display decreased filipin contents when co-cultured with hUCB-MSCs and EEZE together. Furthermore, treatment with EEZE had no effect on the cholesterol accumulation in FB NPC1 cells (Fig. 5a). This result suggests that hUCB-MSC-secreted 14,15-EET might reduce cholesterol accumulation in FB NPC1 cells. To further validate our observation regarding hUCB-MSC treatment in FB NPC1 cells, the direct effect of 14,15-EET on FB NPC1 cells was assessed. As expected, 14,15-EET-treated FB NPC1 cells showed decreased filipin activity. The measured filipin decreased in a dose-dependent manner, which suggests that 14,15-EET decreased unesterified cholesterol accumulation (Fig. 5b).

As previously reported, we identified that hUCB-MSCs lower cholesterol synthesis by reducing the protein expression of the rate-limiting enzyme in cholesterol synthesis^16^. Sterol regulatory element-binding proteins (SREBPs) are known to control lipid synthesis, particularly when these membrane-bound transcription factors become activated and enter the nucleus to activate genes involved in the synthesis and uptake of cholesterol in non-hepatic cells^22^. We measured the cleaved nuclear form of SREBP2 (SREBP2-N) by immunoblot and detected increased levels of SREBP2-N in FB NPC1 cells compared with those in normal human FBs. Decreased levels of SREBP2-N were identified when hUSC-MSCs were indirectly co-cultured with FB NPC1 cells, indicating that hUCB-MSCs lower cholesterol accumulation by reducing cholesterol synthesis, which is consistent with our previous report. EEZE treatment in the co-culture system blocked the effect of hUCB-MSCs by increasing the SREBP2-N levels, which indicates that the therapeutic effect of hUCB-MSCs is partially mediated by 14,15-EET. Significant differences were not identified when EEZE was used alone in FB NPC1 cells (Fig. 5c). The SREBP2 levels were also decreased when FB NPC1 cells were treated with 14,15-EET (Fig. 5d). In conclusion, 14,15-EET decreased the accumulation of unesterified cholesterol in FB NPC1 cells by reducing SREBP2, thus decreasing cholesterol synthesis.

### hUCB-MSCs reduce cholesterol accumulation via autophagic pathway regulation

**Fig. 6:**
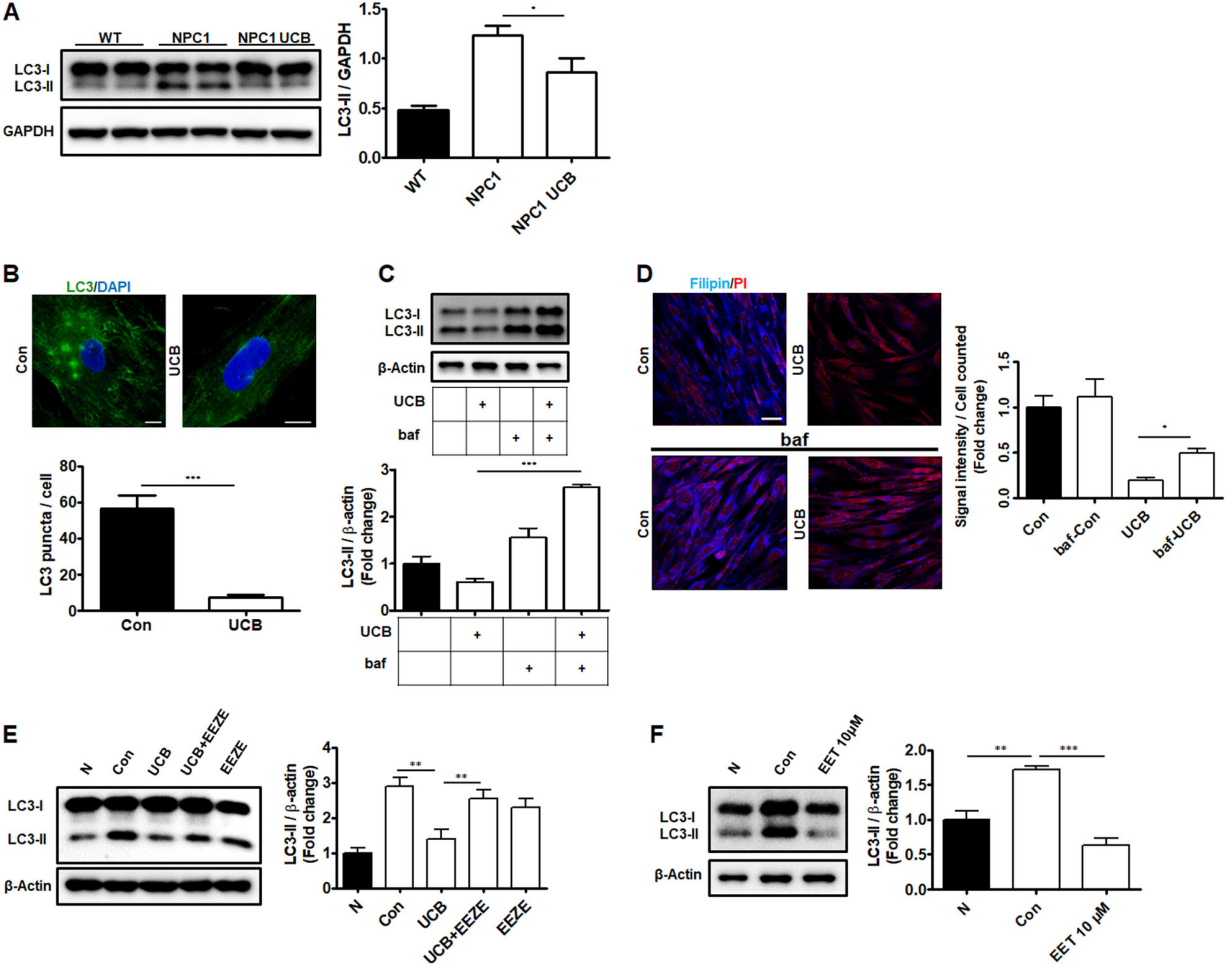
Therapeutic effect of 14,15-EET is regulated by autophagy signaling. **a** Western blot analysis showing the expression pattern of LC3 protein in the cerebella of 8-week-old WT, NPC1-mutant mice (NPC1) and NPC1-mutant mice treated with hUCB-MSCs intranasally (NPC1-UCB). **b** The results of NPC1 patient fibroblasts (Con) and NPC1 patient fibroblasts cultured with hUCB-MSCs (UCB) were compared using immunohistochemistry via staining with LC3. **c** Protein levels of LC3 in NPC1 patient fibroblasts, treated with or without hUCB-MSCs for 72 h, in the presence or absence of 400 nM bafA_1_ for the last 4 h are shown. **d** The results of filipin staining on NPC1 patient fibroblasts (Con), treated with or without hUCB-MSCs for 72 h (UCB), in the presence or absence of 400 nM bafA_1_ for the last 4 h (baf-Con, baf-UCB) are shown. **e** The quantification of LC3 protein was analyzed in normal fibroblasts (N), NPC1 patient fibroblasts (Con), hUCB-MSC-treated NPC1 patient fibroblasts (UCB), hUCB-MSC-co-treated and EEZE-co-treated NPC1 patient fibroblasts (UCB EEZE), and EEZE-treated NPC1 patient fibroblasts (EEZE). **f** LC3 protein levels in normal fibroblasts (N), NPC1 patient fibroblasts (Con), and 14,15-EET-treated (10 μM) NPC1 fibroblasts (EET 10 μM) were also analyzed. Scale bars = 50 μm. All data represent the mean ± SD. *P < 0.05; **P < 0.01; ***P < 0.001

In NPC1 disease, the degradation of autophagosomes during autophagic flux is markedly impaired, leading to LC3-II and p62 accumulation^23^. To examine whether reduced cholesterol levels in NPC1-UCB mice were related to autophagy, we measured the LC3-II levels by immunoblotting. We found that the LC3-II levels were significantly increased in the cerebellum derived from the NPC1-mutant mice, while the LC3-II levels were reduced in the NPC1-UCB mice; the NPC1-mutant mice showed a 2.57-fold increase in the LC3-II expression compared to that of the wild-type mice, while the NPC1-UCB mice only increased 1.79-fold (Fig. 6a). When confirmed with p62, the NPC1-mutant mice showed a 2.31-fold increased expression and the NPC1-UCB mice exhibited a 1.1-fold increased expression compared to the wild-type mice (Fig. S4A). Our results demonstrate that the impaired autophagic pathway in NPC1-mutant mice may be ameliorated through the engraftment of hUCB-MSCs.

To confirm our findings, we co-cultured hUCB-MSCs with FB NPC1 cells in a transwell system and measured the endogenous levels of LC3 using ICC (Fig. 6b). The number of LC3^+^ vesicles in FB NPC1 cells was reduced by 7.2-fold when co-cultured with hUCB-MSCs. In line with the in vivo results shown in Fig. 6a, the LC3-II levels were decreased by 1.4-fold in the hUCB-MSCs co-cultured with FB NPC1 cells compared to those of the controls. This difference was no longer detectable following treatment with the lysosomal inhibitor bafilomycin A1 (bafA) (Fig. 6c). These data suggest that hUCB-MSCs enhance autophagic signaling in NPC1 disease. To further assess the cholesterol levels in vitro, we conducted a filipin assay. We found that the filipin intensity was decreased by 5.0-fold when FB NPC1 cells were co-cultured with hUCB-MSCs. Importantly, when autophagic flux was blocked by bafA_1_, the intracellular cholesterol levels were increased compared with those of the bafA_1_-non-treated group (Fig. 6d). Thus, we inferred that the regulation of cholesterol by hUCB-MSCs is partially mediated by increased autophagic signaling in NPC1-mutant subjects.

The improved autophagic degradation in NPC1-UCB mice encouraged us to analyze whether hUCB-MSCs affect autophagy activity in FB NPC1 cells. Similar to that in the cerebella of NPC1-mutant mice, the LC3-II levels were increased in the FB NPC1 cells compared to those in the normal human FBs. As expected, when FB NPC1 cells were co-cultured with hUCB-MSCs, the enhanced accumulation of LC3-II was significantly reduced. These effects of hUCB-MSCs were confirmed by Western blot analysis of p62 and presented less accumulation when hUCB-MSCs are were co-cultured with FB NPC1 cells (Fig. S4B). The reduced LC3-II was increased when cultured with EEZE. Because no changes were identified when EEZE was used alone, we concluded that 14,15-EET secreted from hUCB-MSCs rescues autophagy defects in FB NPC1 cells. Western blot analysis showed a 2.89-fold increase in the LC3-II levels in the FB NPC1 cells compared with those in the normal human FBs. In addition, a 1.3-fold increase in the LC3-II levels was identified when FB NPC1 cells were co-cultured with hUCB-MSCs, while a 2.53-fold increase in LC3-II was identified when EEZE was added to the co-culture system (Fig. 6e). In addition, to identify activated autophagic reactions during the co-culture of hUCB-MSCs and FB NPC1 cells, the mRNA levels of autophagy-related genes were analyzed. The *ATG-9*, *BECLIN*, and *ATG-5* levels were increased by 4.80-fold, 3.15-fold, and 2.31-fold, respectively, during co-culture. However, when EEZE was added to the co-culture, *ATG-9* and *BECLIN* only increased by 1.8-fold and 1.5-fold, respectively, while *ATG-5* was decreased 0.58-fold (Fig. S5). Because cholesterol is reduced in 14,15-EET-treated FB NPC1 cells, LC3-II is most likely reduced in the same manner. To evaluate this finding, immunoblotting was performed on 14,15-EET-treated FB NPC1 cells. Reduced LC3-II accumulation was identified in 14,15-EET-treated FB NPC1 cells (Fig. 6f).

## Discussion

In this study, we have explored the non-invasive strategy of intranasally delivering hUCB-MSCs to treat NPC1 disease. The administration of hUCB-MSCs improved the functional outcome and attenuated the pathological features of NPC1-mutant mice. The intranasal delivery of hUCB-MSCs increased the motor function of NPC1-mutant mice, and this phenomenon may be explained by the increased Purkinje cell survival. Furthermore, the therapeutic effects of hUCB-MSCs in NPC1-mutant mice were clarified by the observation of decreased cholesterol accumulation and corrected impaired autophagy signaling in NPC1-mutant mice and NPC1 patient FBs. These results demonstrate that intranasal delivery could be a suitable method for stem cell transplantation in NPC1 patients.

The assessment of intranasal delivery of cell-based therapy remains in the early phase. Regarding migration, the actual pathways by which cells migrate or the mechanism of the movement have not been fully explained. Although neural precursor cells that originate from the brain have been shown to migrate along the RMS^24^, intranasally delivered MSCs have been suggested to migrate into the brain along the olfactory and trigeminal nerves via the parenchymal and cerebrospinal fluid pathways^11^. A study regarding the SVZ suggested that intranasally delivered MSCs were not observed near the SVZ, postulating that MSCs at the lesion induced neurotrophic factor production in the SVZ, potentially through paracrine signaling by MSCs^25^. In this study, GFP-expressing hUCB-MSCs were detected on the RMS path near the SVZ after 24 h of administration, showing that hUCB-MSCs can migrate throughout the peripheral olfactory system connecting the nasal passages with the OB and rostral brain regions.

Another question addressed is whether intranasally delivered hUCB-MSCs can target cells to the lesion area of NPC1-mutant mice. A hallmark of NPC1 disease in both humans and mouse models is cerebellum involvement, which is clinically manifested by tremors and ataxia^18^. As the disease progresses, neurodegeneration is also apparent in some brain regions, particularly in the Purkinje cells of the cerebellum^26^. In NPC1 patients, the neurological symptoms are well established to be caused by degeneration and the loss of Purkinje cells^27^. Because the cerebellum is a critical hallmark of NPC1 disease, it is crucial to determine whether hUCB-MSCs migrate to the cerebellum from the nasal cavity. In a recent report, [^3^H]thymidine-labeled MSCs were intranasally delivered, and the highest percentage of cells appeared in the cortex, cerebellum, brain stem, and spinal cord 4 h after application^12^. However, in our study, only one GFP-positive cell was identified in the region of the CB nuclei and the HP. Therefore, the therapeutic effects of hUCB-MSCs in cerebella are thought to be regulated by the local delivery of cytokines rather than direct cell-to-cell interactions.

In the previous decade, MSCs have been utilized in various disease models. From these studies, one outstanding observation was that MSCs were frequently associated with functional improvements; however, there was no evidence of cell engraftment^28^. This finding suggests that the therapeutic effects of MSCs are caused by releasing growth factors and other molecules rather than by using their stem cell-like ability to differentiate or fuse with existing cells^29^. BM-MSC-derived CCL2 cells have been suggested to be an effective adjuvant for improving neurogenic effects and a critical factor for the increased proliferation and neural differentiation of NPC1 neural stem cells^8^. An NPC1-mutant mouse study concluded that VEGF released from BM-MSCs can reduce pathological changes by increasing autophagic degradation; moreover, the authors of this study suggested a novel pathogenic mechanism in NPC1 disease, indicating a potential therapeutic approach via the VEGF/SphK pathway^30^. In the current findings, we present a novel molecule, 14,15-EET, secreted from hUCB-MSCs as being able to ameliorate the accumulation of unesterified cholesterol by inducing changes in autophagy function in NPC1 disease. We believe 14,15-EET could be a therapeutic candidate for NPC1 disease treatment.

Arachidonic acid is metabolized by CYP to biologically activate eicosanoids, which are critical regulators of numerous biological processes^31^. The expression of the CYP family has been well characterized in both the brain and associated vasculature^32^, particularly CYP epoxygenase enzymes from the CYP2C and CYP2J subfamilies, which metabolize arachidonic acids to biologically active EETs^33^. Mice fed atherogenic diets tend to have significantly higher hepatic cholesterol levels when hepatic CYP epoxygenase metabolic activity is suppressed, which indicates that the decreased expression levels of *Cyp2C37*, *Cyp2C40*, *Cyp2C54*, *Cyp2J5*, and *Cyp2J11* correlate with the decreased levels of 14,15-EET formation in the liver^34^. Previously, quantitative real-time PCR was performed on mRNA isolated from 9-week-old female NPC1-mutant mouse livers to validate the decreased expressions of *Cyp2C37*, *Cyp2C40*, and *Cyp2C54*^35^. 14,15-EET is an arachidonic acid metabolite synthesized by CYP epoxygenases of the 2C and 2J subfamilies^36^. In neurons, 14,15-EET is known for its neuroprotective effects on oxidative stress^37^. The overexpression of CYP2J2 in mice showed significantly decreased plasma triglyceride levels and lipid accumulation in the liver, improved liver function, reduced inflammatory responses, and reduced hepatic oxidative stress compared to the WT mice^20^. Another study of CYP2J2-overexpressing mice showed significantly decreased plasma and liver triglyceride levels, decreased liver cholesterol levels, and decreased high-fat-diet-induced lipid levels^38^.

Because of these properties, we aimed to address whether 14,15-EET has a therapeutic effect on NPC1 disease. By demonstrating the positive effect of 14,15-EET on cholesterol levels in NPC1 patient FBs and that the expression of genes in the CYP2C family was decreased in NPC1-mutant mice, the neuropathology of NPC1 disease may partially be a result of the altered expression of genes in the CYP family in the brain. It is known that 14,15- EET promotes cell proliferation and survival by alleviating the apoptosis pathway^39^. Furthermore, a study regarding 14,15-EET strongly suggests that 14,15-EET-mediated protective effects involve modulation of the autophagic response, which, in turn, promotes cell survival in cardiac cells during starvation^40^. Autophagy is a critical factor in NPC1 disease, and impaired autophagic flux in NPC1 disease is associated with decreased autophagosome–lysosome fusion, which leads to Purkinje cell loss^30^. The current data show that 14,15-EET is released from hUCB-MSCs, which could reduce the pathological changes associated with NPC1 disease by increasing autophagic signals. The mRNA levels of the autophagy signal markers *BECLIN*, *ATG-5*, and *ATG-9* were decreased by co-treatment with EEZE, which suggests that 14,15-EET affects the autophagic pathway. Taken together, we hypothesize that 14,15-EET may be dysregulated in NPC1 disease and linked to defects in LE/L cholesterol efflux.

In conclusion, we have implemented a non-invasive strategy to treat NPC1-mutant mice with hUCB-MSCs, thus presenting a new therapeutic treatment for NPC1 patients. The findings in which the accumulation of cholesterol was ameliorated and motor function was improved in NPC1-mutant mice suggest that intranasally delivered hUCB-MSCs have a therapeutic effect on neurological disorders. We have shown that hUCB-MSCs can decrease cholesterol levels in NPC1 patient-derived FBs, and we believe this phenomenon is regulated by the epoxyeicosatrienoic acid 14,15-EET. These findings illustrate a non-invasive route for hUCB-MSC delivery, which may be a viable therapeutic treatment option for NPC1 patients.

## Electronic supplementary material

Supp figure legends

Supp 1

Supp 2

Supp 3

Supp 4

Supp 5
