# Supplementary material for: Stem cell-secreted 14,15- epoxyeicosatrienoic acid rescues cholesterol homeostasis and autophagic flux in Niemann–Pick-type C disease
Source: Exp Mol Med. 2018 Nov 14;50(11):149. doi: 10.1038/s12276-018-0176-0 (PMC6235958; doi:10.1038/s12276-018-0176-0)
Supplement: Supplementary file 1 — Supp figure legends [file 12276_2018_176_MOESM1_ESM.docx]

**Supplementary materials**

**Figure S1. Detection of GFP-expressing hUCB-MSCs in the brain of mice 24 hour after intranasal administration.** (A) Section stained with 4’,6-diamidino-2-phenylindole (DAPI) nuclear staining (blue) show the presence of GFP positive hUCB-MSCs (green) in Olfactory bulb (OB), subventricular zone (SVZ), hippocampus (HP) and cerebellar (CB). (B) Flow cytometric analysis was performed in dissected and suspended brain areas (OB, SVZ, HP, CB) and (%) of GFP positive hUCB-MSCs were quantified (n=3). Scale bars = 100 μm.

**Figure S2. Motor function in NPC1-mutant mice (Male and Female, NPC1) after intranasal administration of hUCB-MSCs (NPC1-UCB)** All data represent the mean ± SD. **, P < 0.01; ***, P < 0.001.

**Figure S3.** **Increased survival rate of Purkinje neurons after hUCB-MSC administration** (A) Cresyl violet staining of the anterior region of the cerebella of NPC1-mutant mice (NPC1) show increased survival rate of Purkinje neurons after hUCB-MSCs were administrated intranasally (NPC1 UCB). (B) The posterior region of the cerebellum was examined and showed the same result. Scale bars = 50 μm. All data represent the mean ± SD. ***, P < 0.001.

**Figure S4 Therapeutic effect of hUCB-MSC is regulated by autophagy signaling** (A) Western blot analysis showing the expression pattern of p63 protein in the cerebella of 8-week-old WT, NPC1-mutant mice (NPC1) and NPC1-mutant mice treated with hUCB-MSC intranasally (NPC1 UCB). (B) The expression level of p63 protein was analyzed in normal fibroblasts (N), NPC1 patient fibroblasts (Con), hUCB-MSC treated NPC1 patient fibroblasts (UCB) by western blot.

**Figure S5. hUCB-MSC reduces cholesterol through autophagy signaling** mRNA levels of autophagy markers (*ATG9, BECLIN,* and *ATG5*) were analyzed in NPC1 patient fibroblasts as the control (Con), fibroblasts co-cultured with hUCB-MSC (UCB), and a co-culture system treated with EEZE (UCB EEZE). Additionally, relative gene expression levels were quantified compared to NPC1 patient fibroblasts, in which expression is considered to be 1 for all genes. All data represent the mean ± SD. **, P < 0.01;
