# Supplementary figures and images for: Stem cell-secreted 14,15- epoxyeicosatrienoic acid rescues cholesterol homeostasis and autophagic flux in Niemann–Pick-type C disease
Source: Exp Mol Med. 2018 Nov 14;50(11):149. doi: 10.1038/s12276-018-0176-0 (PMC6235958; doi:10.1038/s12276-018-0176-0)

**A**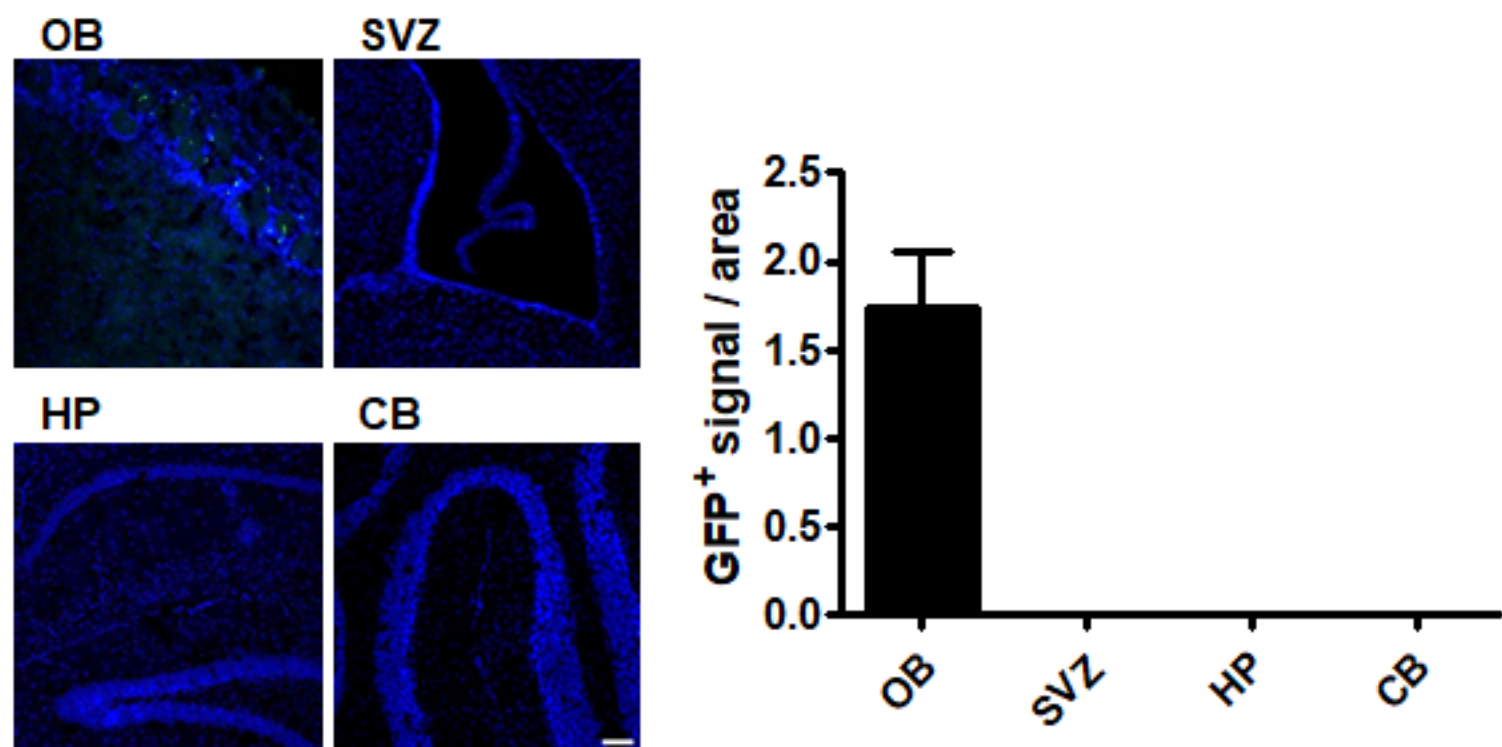**B**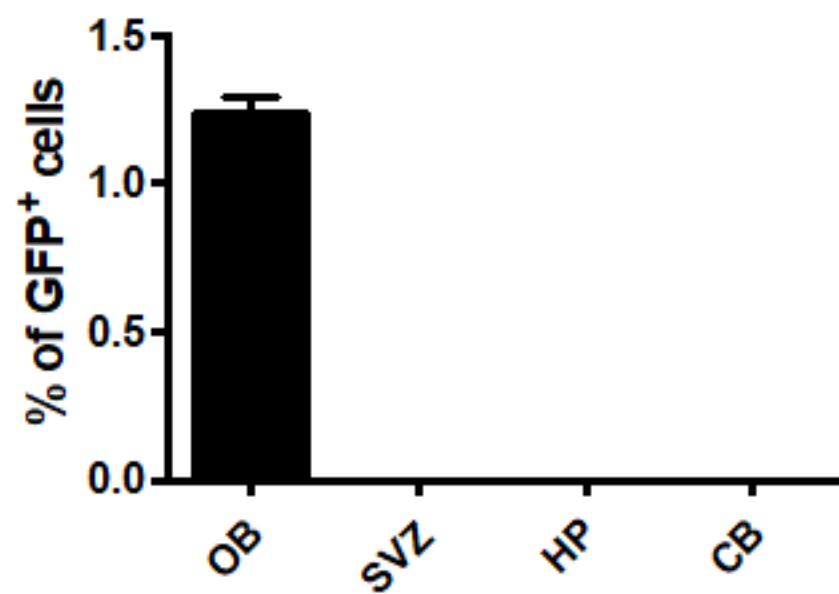

Supplement: Supplementary file 2 — Supp 1 [file 12276_2018_176_MOESM2_ESM.pdf]

## Males + females

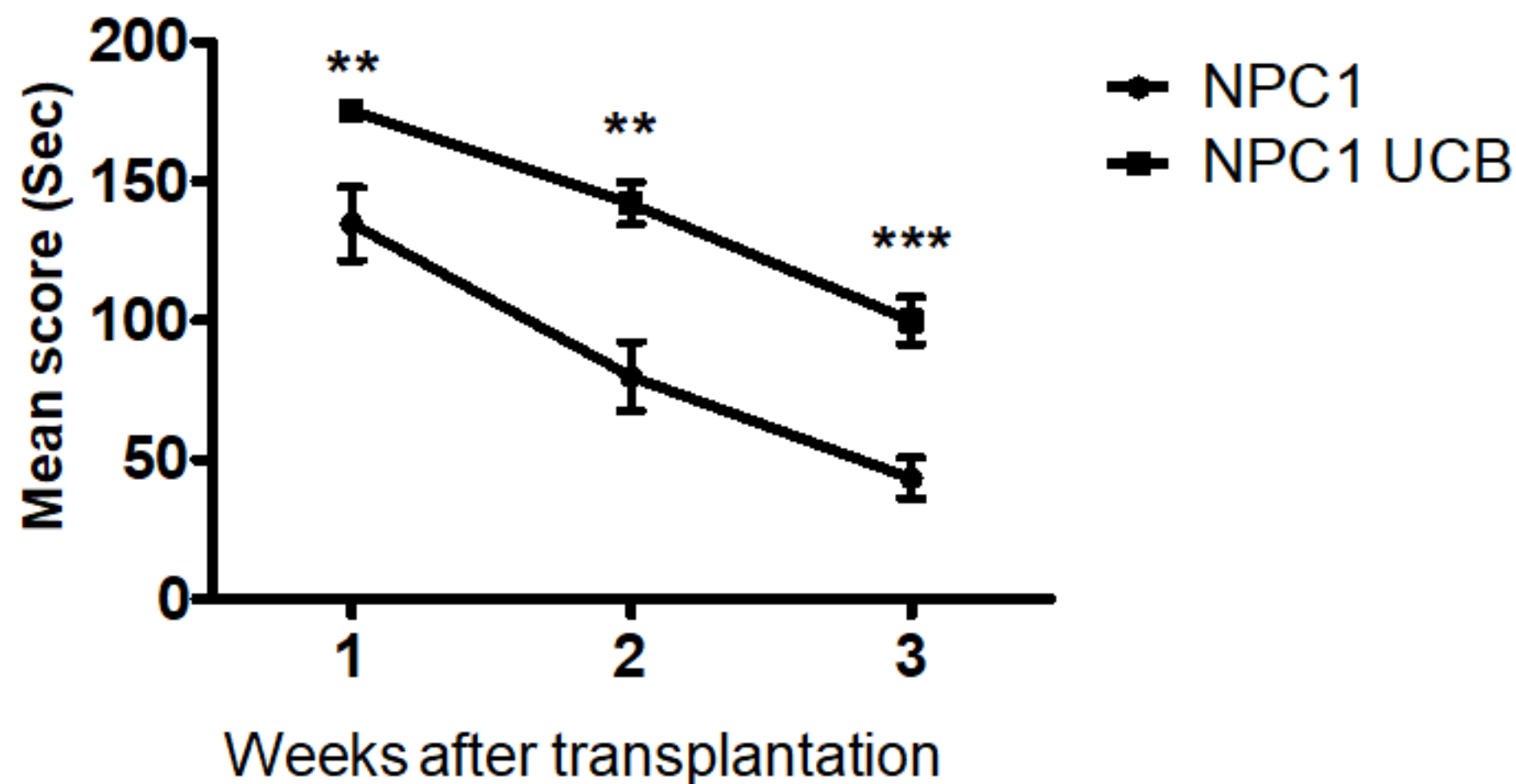

Supplement: Supplementary file 3 — Supp 2 [file 12276_2018_176_MOESM3_ESM.pdf]

**A**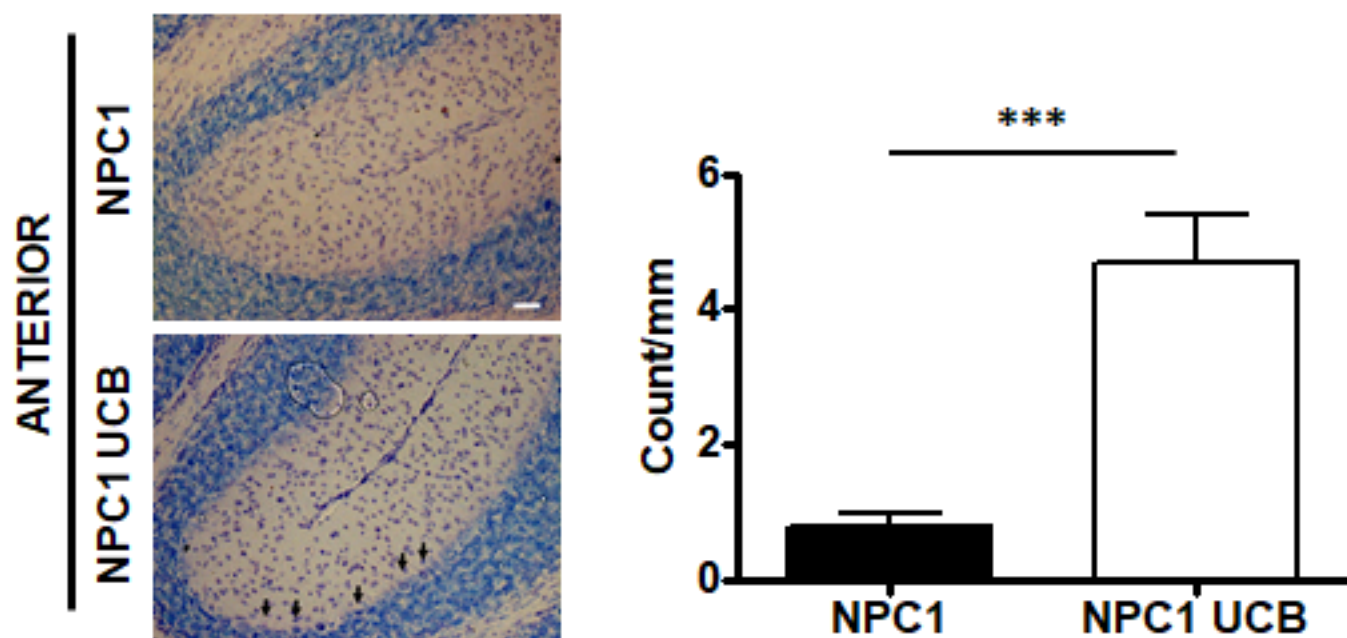**B**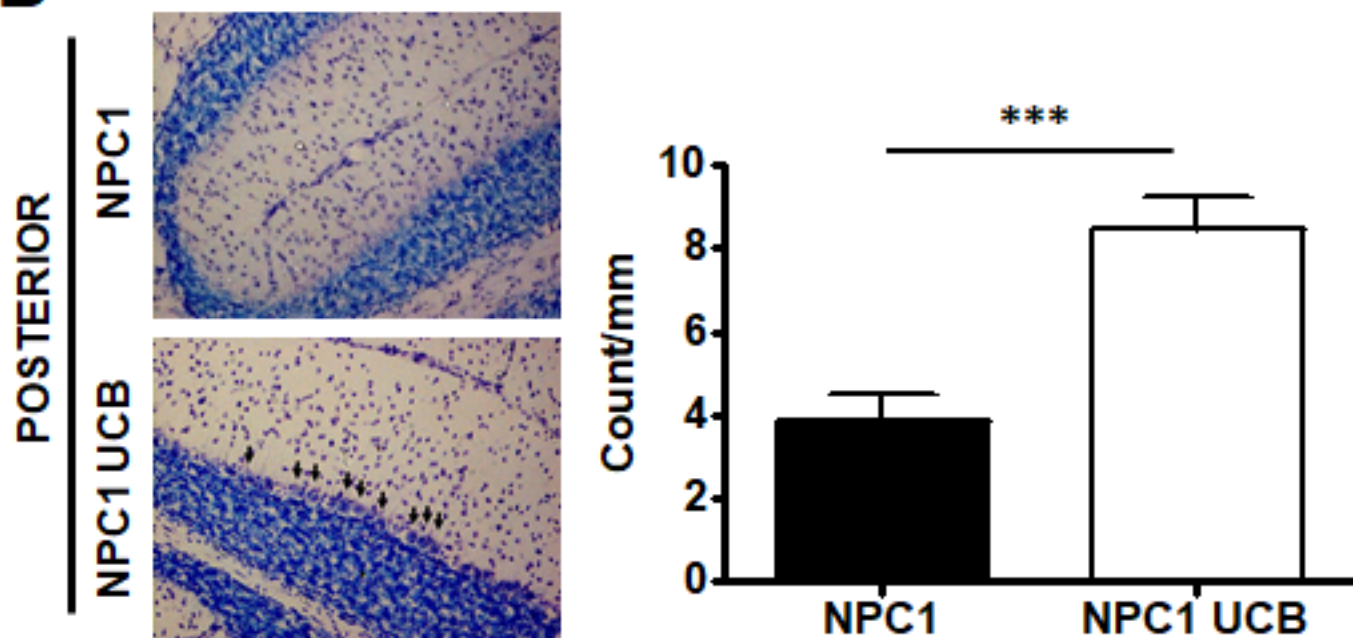

Supplement: Supplementary file 4 — Supp 3 [file 12276_2018_176_MOESM4_ESM.pdf]

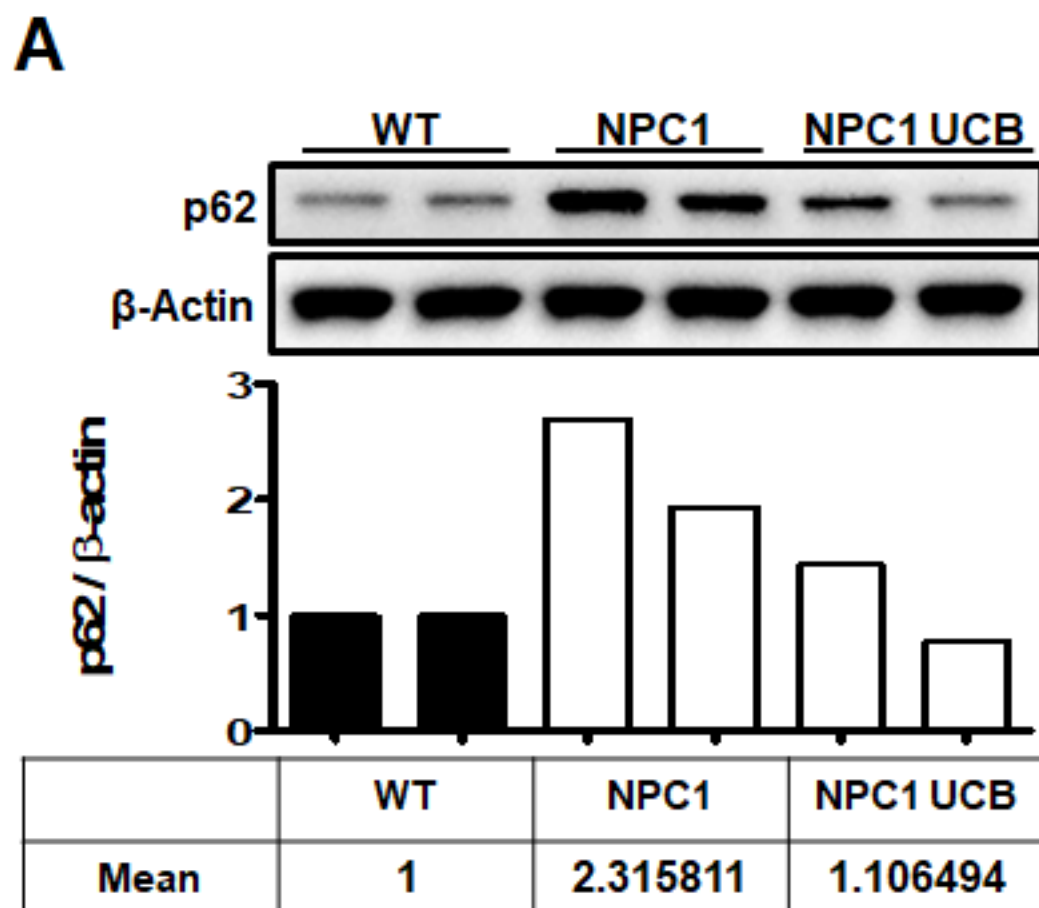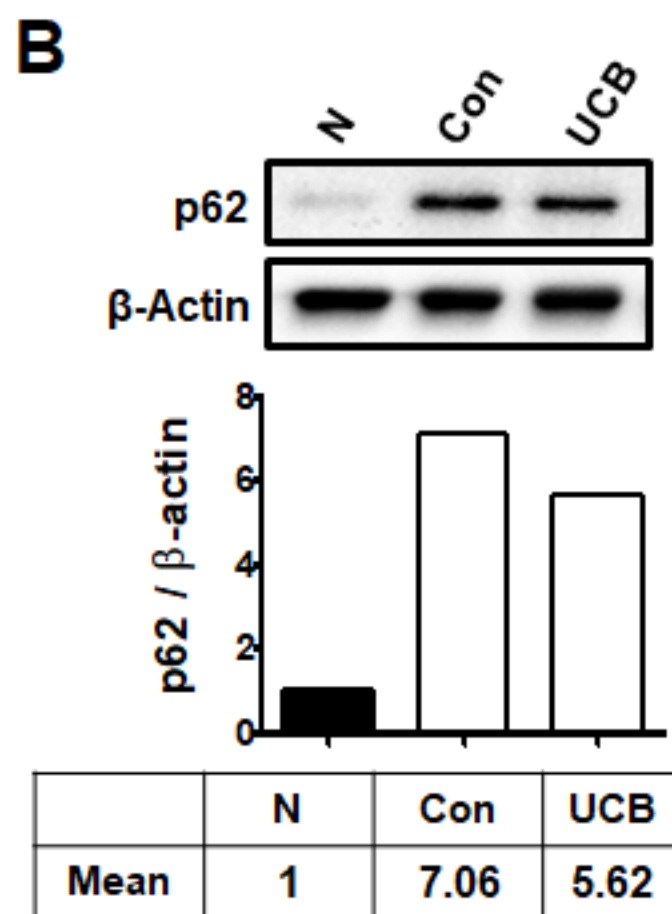

**Kang et al. Suppl. Figure 4**

Supplement: Supplementary file 5 — Supp 4 [file 12276_2018_176_MOESM5_ESM.pdf]

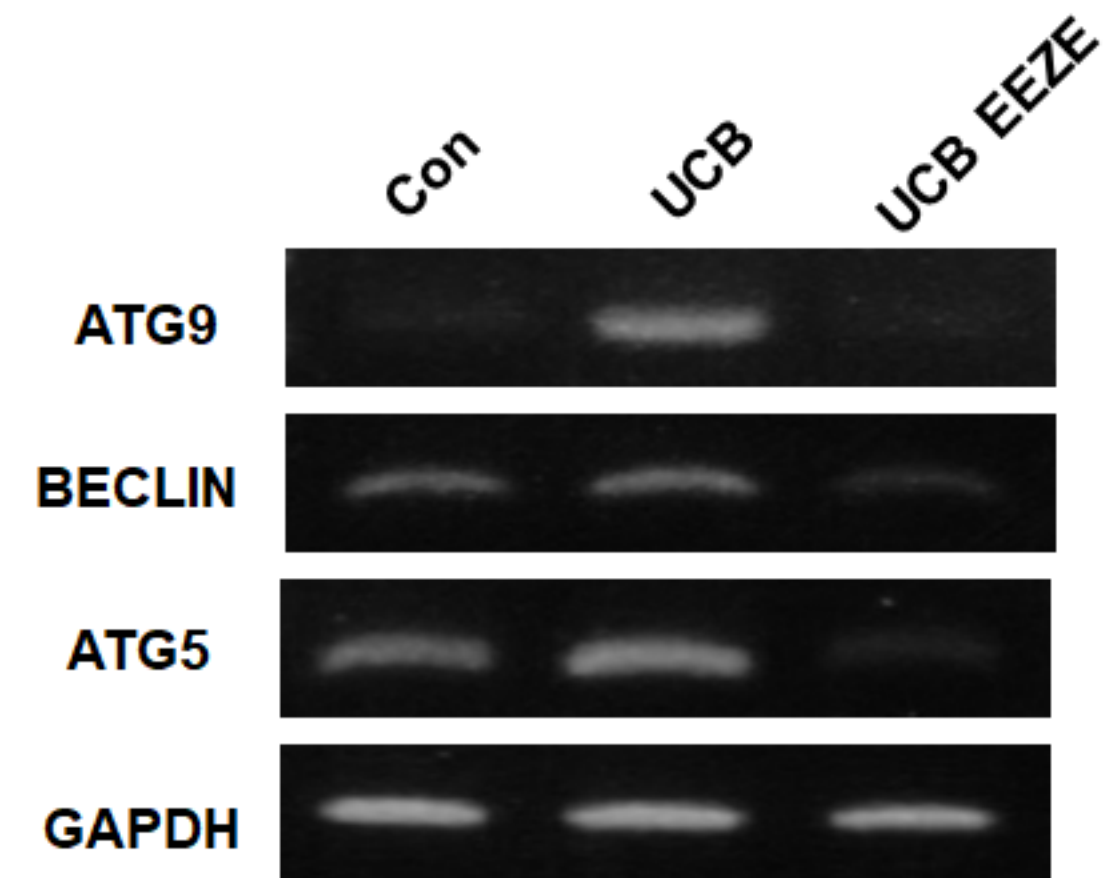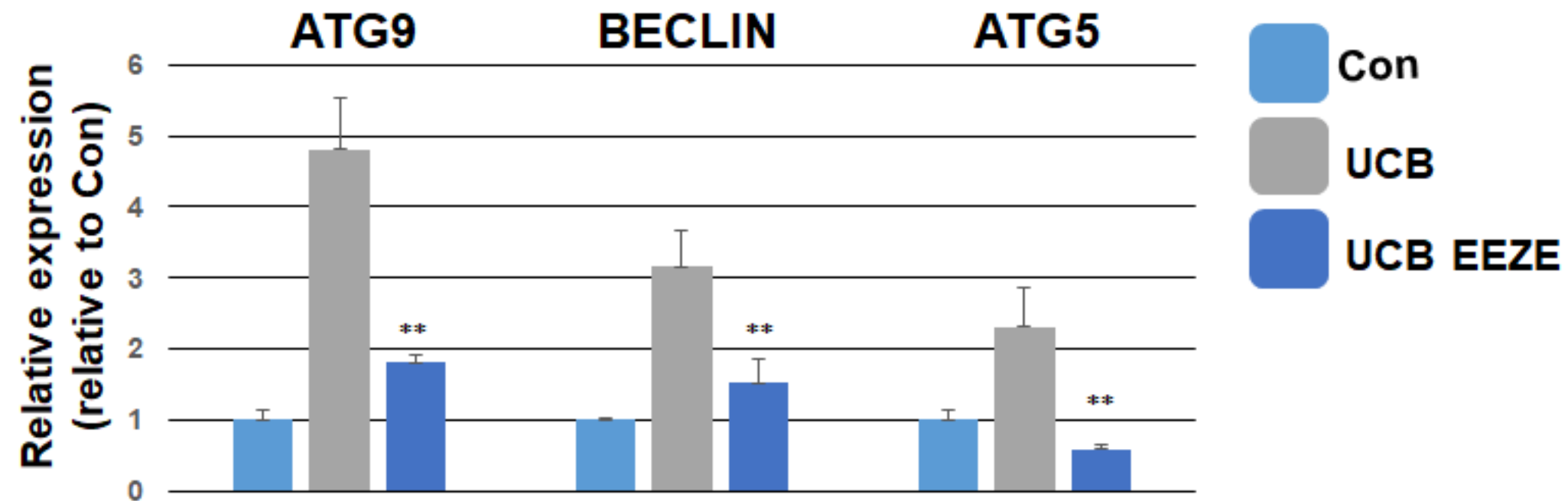

Kang *et al.* Suppl. Figure 5

Supplement: Supplementary file 6 — Supp 5 [file 12276_2018_176_MOESM6_ESM.pdf]
